# Well-Defined Diblock Poly(ethylene glycol)-*b*-Poly(ε-caprolactone)-Based Polymer-Drug Conjugate Micelles for pH-Responsive Delivery of Doxorubicin

**DOI:** 10.3390/ma13071510

**Published:** 2020-03-26

**Authors:** Amin Jafari, Lingyue Yan, Mohamed Alaa Mohamed, Yun Wu, Chong Cheng

**Affiliations:** 1 Department of Chemical and Biological Engineering, University at Buffalo, The State University of New York, Buffalo, NY 14260, USA; aminjafa@buffalo.edu (A.J.); mm446@buffalo.edu (M.A.M.); 2 Department of Biomedical Engineering, University at Buffalo, The State University of New York, Buffalo, NY 14260, USA; lingyuey@buffalo.edu; 3 Chemistry Department, Faculty of Science, Mansoura University, Mansoura, 35516, Egypt

**Keywords:** polymer-drug conjugate, drug delivery, diblock copolymer, doxorubicin, poly(ε-caprolactone), poly(ethylene glycol), ring-opening polymerization, CuAAC click reaction, Schiff base reaction, micelle

## Abstract

Nanoparticles have emerged as versatile carriers for various therapeutics and can potentially treat a wide range of diseases in an accurate and disease-specific manner. Polymeric biomaterials have gained tremendous attention over the past decades, owing to their tunable structure and properties. Aliphatic polyesters have appealing attributes, including biodegradability, non-toxicity, and the ability to incorporate functional groups within the polymer backbone. Such distinctive properties have rendered them as a class of highly promising biomaterials for various biomedical applications. In this article, well-defined alkyne-functionalized poly(ethylene glycol)-*b*-poly(ε-caprolactone) (PEG-*b*-PCL) diblock copolymer was synthesized and studied for pH-responsive delivery of doxorubicin (DOX). The alkyne-functionalized PEG-*b*-PCL diblock copolymer was prepared by the synthesis of an alkyne-functionalized ε-caprolactone (CL), followed by ring-opening polymerization (ROP) using PEG as the macroinitiator. The alkyne functionalities of PEG-*b*-PCL were modified through copper(I)-catalyzed alkyne-azide cycloaddition (CuAAC) click reaction to graft aldehyde (ALD) groups and obtain PEG-*b*-PCL-*g*-ALD. Subsequently, DOX was conjugated on PEG-*b*-PCL-*g*-ALD through the Schiff base reaction. The resulting PEG-*b*-PCL-*g*-DOX polymer-drug conjugate (PDC) self-assembled into a nano-sized micellar structure with facilitated DOX release in acidic pH due to the pH-responsive linkage. The nanostructures of PDC micelles were characterized using transmission electron microscopy (TEM) and dynamic light scattering (DLS). In vitro studies of the PDC micelles, revealed their improved anticancer efficiency towards MCF-7 cells as compared to free DOX.

## 1. Introduction

Cancer is the second cause of death in the United States. The treatment option depends on various factors, including the type and stage of cancer and patient preference. Chemotherapy is a type of treatment for many types of cancer, using anticancer drugs to stop or slow the growth of cancer cells. In order to achieve better chemotherapeutic efficacy, the innovative design of drug delivery systems for cancer treatment has attracted considerable attention over the past decades [1,2]. The employment of nanotechnology in anticancer drug delivery systems has resulted in significant innovations in this field. Nanomedicine, the integration of medicine with nano-sized vector, has emerged for the treatment of cancer diseases that can carry drugs or therapeutic agents and release them in a controlled fashion at the desired site of action. Despite extensive study of such systems in past decades, there are challenging design parameters that need to be addressed carefully, such as blood circulation, nonspecific interaction, degradation, solubility, and kidney clearance. Accordingly, polymeric nanocarriers have emerged as promising biomaterials that may overcome biological barriers, protect the therapeutic cargo, and effectively deliver them to the diseased tissue. Among the varieties of anticancer polymeric nanomedicines that have been reported, biodegradable polymer-drug conjugates (PDCs) can potentially lead to sustained drug delivery without burst release, and their long-term side effect is minimal [2,3,4,5].

**Figure 1:**
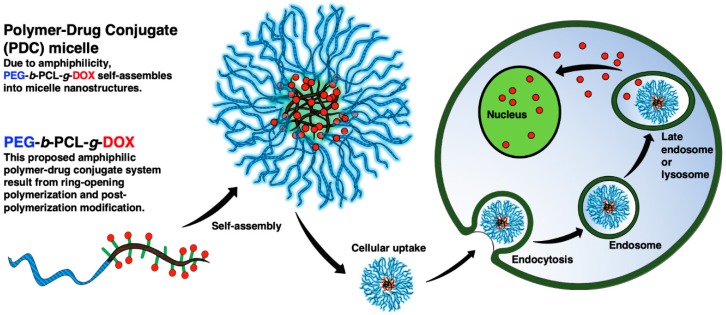
Conceptual schematic of anticancer drug delivery using PEG-*b*-PCL-*g*-DOX PDC micelle.

The idea of the coupling of drug molecules to a polymer was first introduced by Ringsdorf in 1975, whereby a spacer was used as a linkage between the drug and the polymer [6]. Based on the specific design of the spacer, the drug can be released at the particular site of interest, i.e., tumor tissue for the case of cancer treatment. Since then, a wide variety of PDCs have been studied, and several of them have been utilized successfully in clinical research [7]. An ideal PDC can demonstrate several advantages over free drugs, including less significant side effects, enhanced therapeutic efficacy, ease of drug administration, and improved patient compliance. Amphiphilic block copolymers have been comprehensively investigated since they can self-assemble into micelle structure and carry therapeutics by either physical encapsulation or covalent conjugation [8,9,10]. PDC micelles derived from amphiphilic diblock PDCs may be able to achieve the features mentioned above. The active drug is conjugated within the hydrophobic block in the core domains of micelles, protected from the surrounding environment. The hydrated outer shells consisting of the hydrophilic polymer block can provide desirable properties related to the systemic circulation and interaction with the surrounding environment. Hence, PDC micelles can serve appropriately as drug nanocarriers. Figure 1 illustrates the conceptual schematic for anticancer drug delivery using PDC micelles.

Some of the properties of polymeric micelles are associated with the type of the hydrophobic block of the PDC. These properties include micelle stability, drug loading capacity, and drug release profile [8]. A broad range of polymers can serve for the hydrophobic block, such as poly(propylene glycol) (PPO) [11], poly(amino acid) with functional groups [12], and aliphatic polyesters, including poly(lactic acid) (PLA) [13] and poly(ε-caprolactone) (PCL) [14,15]. Among these polymers, aliphatic polyesters have been widely studied and investigated due to their remarkable biodegradability [16,17,18].

In this study, PCL was used as the hydrophobic block of PDC, which formed drug-conjugated micelles for pH-responsive delivery of doxorubicin (DOX). PCL is composed of hexanoate repeat units. As an FDA-approved polymer for clinic use, PCL has been utilized in a broad range of biomedical applications, such as drug and gene delivery, implants, stents, and prosthetics [19,20,21,22,23]. All of these notable applications rely on the biodegradable feature of PCL [22]. In vivo degradation of PCL leads to nontoxic 6-hydroxyhexanoic acid, which is a natural human body metabolite [24]. Unfavorable high acidity polymer degradation products may be hazardous to human health and also deactivate the loaded drugs. The degradation products of PCL have less acidity compared to the degradation products of other types of aliphatic polyesters, such as PLA and polyglycolide (PGA) [25]. Therefore, PCL can be considered as the preferred choice of aliphatic polyester for biomedical applications and therapeutic delivery applications. PCL itself without modification cannot be directly employed for drug delivery applications via systemic administration due to its hydrophobic and semi-crystalline nature. For this reason, the incorporation of PCL with a robust hydrophilic polymer or other small molecules is a necessary strategy. Poly(ethylene glycol) (PEG) with its distinct properties in the diblock PCL-*b*-PEG copolymers form, can overcome the limitations of PCL and serve as a promising biomaterial for therapeutic delivery applications [8,10,15,26]. The amphiphilic nature of PEG-*b*-PCL copolymers endows them with surfactant properties, enabling them to self-assemble into micellar structures. When PEG-*b*-PCL is employed in an aqueous environment, hydrophilic PEG blocks enrich at the shell of the micelles. Such a PEGylated surface can minimize premature renal clearance from the blood and prevent nonspecific interactions.

The main pathway for synthesis of PEG-*b*-PCL is the ring-opening polymerization (ROP) of ɛ-caprolactone (CL) using PEG as the macroinitiator [17,18,27,28]. Towards the conjugation of DOX, alkyne-functionalized CL monomer was synthesized in the first step [16]. The alkyne-functionalized PEG-*b*-PCL was further reacted with a pH-responsive spacer molecule through a copper(I)-catalyzed alkyne-azide cycloaddition (CuAAC) click reaction [29,30], tethering aldehyde functionalities with the PCL backbone (PEG-*b*-PCL-*g*-ALD). Subsequently, successful DOX conjugation on PCL was achieved with Schiff base chemistry [31,32]. Representing a novel type of PDC with promising properties, the resulting PEG-*b*-PCL-*g*-DOX was characterized to verify its well-controlled structures. Biomedical assessments were further conducted to reveal the merits of the PDC micelles as a candidate for anticancer nanomedicine. It should be noted that Danafar et al. reported previously the studies of PDC micelles with PEG-*b*-PCL diblock copolymer carrying a conjugated doxorubicin moiety at polymer ω-chain-end with a hydrolyzable ester bond [10]. Based on the chemistry innovation of using a functional CL monomer, our current work enables side-chain functionalization of a PEG-*b*-PCL-based diblock copolymer to conjugate many DOX moieties per macromolecule with acid-labile benzyl imine linkages, leading to a much higher drug loading amount, which is critically important for a drug delivery system.

## 2. Materials and Methods 

### 2.1. Measurements

All the chemical structures were verified at 500 MHz using a Varian INOVA-500 (Varian, Inc., Palo Alto, CA, USA) spectrometer maintained at 25 °C with tetramethylsilane (TMS) as an internal reference standard. The raw data were analyzed using VnmrJ software (v. 4.2, Agilent Technologies, Inc., Santa Clara, CA, USA). 

The progress of ROP and polymer molecular weight distribution was determined using a Viscotek Gel permeation chromatography (GPC) system (Malvern Panalytical, Malvern, UK) equipped with a VE-3580 refractive index (RI) detector, a VE-1122 pump, and two mixed-bed organic columns (PAS-103M and PAS-105M). *N*,*N′*-Dimethylformamide (DMF) containing 0.01 M LiBr was used as the mobile phase with a flow rate of 0.5 mL/min at 55 °C. The calibration curve for the GPC system was obtained using narrowly dispersed linear polystyrene standards [peak-average molecular weight (M_p_) = 0.58, 1.53, 3.95, 10.21, 29.51, 72.45, 205, 467, 1319, and 2851 kDa] purchased from Varian (Varian, Inc., Palo Alto, CA, USA). 

The hydrodynamic diameter (*D*_h_) and the volume-average size distribution of micellar nanoparticles were determined using dynamic light scattering (DLS) on a Zetasizer nano-ZS90 (Malvern Panalytical, Malvern, UK) in water at 25 °C.

The morphology of the micellar nanoparticles was investigated with transmission electron microscopy (TEM) using a JEOL 2010 microscope (JEOL Ltd., Akishima, Japan). The TEM grids (400 mesh carbon-coated B copper) were dip-coated with PDC micelle solution with a DLS count rate of ~250 kcps in water. The water was completely dried under vacuum and no staining agent was applied on the grids.

### 2.2. Materials

1,5,7-Triazabicyclo[4.4.0]dec-5-ene (TBD), doxorubicin hydrochloride (DOX.HCl), and methoxy poly(ethylene glycol) (mPEG_45_-OH; MW ~2 kDa, flakes) were purchased from Sigma-Aldrich. Toluene (HPLC), hexane (HPLC), dichloromethane (DCM; HPLC), chloroform (HPLC), acetone (HPLC), tetrahydrofuran (THF, HPLC), ethyl acetate (HPLC), DMF (HPLC), and diethyl ether (HPLC) were purchased from Fisher Scientific.

Toluene was refluxed over calcium hydride (CaH_2_) for two hours prior to distillation. THF was dried by distillation over sodium-benzophenone when the solution became dark blue. mPEG_45_-OH was dried using azeotropic distillation in dry toluene at 175 °C (2×). All other chemicals were used without further purification.

### 2.3. Synthesis of Alkyne-Functionalized CL (**1**)

**Figure 2:**
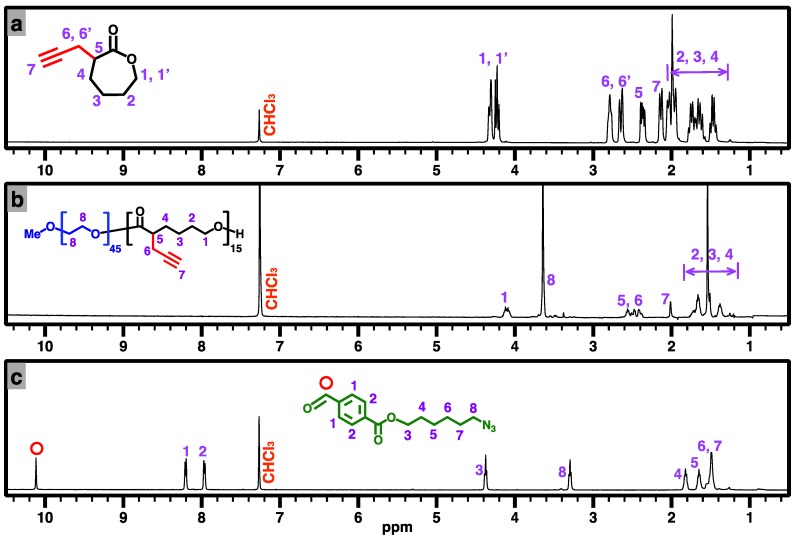
500 MHz ^1^H NMR spectra: (**a**) alkyne-functionalized ε-caprolactone (CL) (**1**), (**b**) alkyne-functionalized poly(ethylene glycol)-*b*-poly(ε-caprolactone) (PEG-*b*-PCL) (**2**), and (**c**) 6-azidohexyl 4-formylbenzoate pH-responsive spacer molecule (**3**) in CDCl_3_.

**Scheme 1:**
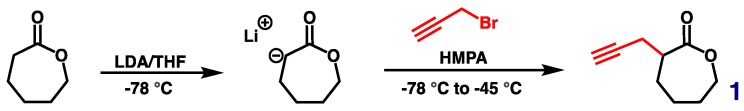
Synthesis of alkyne-functionalized CL (**1**).

Alkyne-functionalized CL (**1**) was synthesized according to Scheme 1, based on a previously reported procedure but with allyl bromide replaced by propargyl bromide [22]. The structure of **1** was confirmed using 500 MHz ^1^H NMR analysis in CDCl_3_ (Figure 2a).

### 2.4. Synthesis of Alkyne-Functionalized Poly(ethylene glycol)-b-poly(ε-caprolactone) (PEG-b-PCL, **2**)

Alkyne-functionalized CL (**1**) monomer was polymerized using the ROP technique with TBD as the organocatalyst [17,22,23,28,33]. Due to the water-sensitive nature of this reaction, all the reagents must be adequately dried. The reaction was conducted in a tightly sealed 10-mL Schlenk flask with a magnetic stirring bar, which was flamed dried under vacuum and refilled with nitrogen (3×). A preweighted amount of dried mPEG_45_-OH macroinitiator (188 mg, 93 µmol) and TBD (46 mg, 328 µmol, 5 mol% relative to **1**) were dissolved in dry toluene (10 mL) and added to the flask via a nitrogen-purged syringe. The solution was stirred for 30 min at room temperature. Monomer **1** (1000 mg, 6.57 mmol) was dissolved in dry toluene (10 mL) and added to the flask with an air-free syringe to start the reaction at room temperature. The ROP process was carefully monitored using GPC to check MW and MW distribution of the formed polymer and using ^1^H NMR characterizations to determine monomer conversion. Once the desired monomer conversion was achieved, the reaction was quenched by adding a few drops of acetic acid. The polymer was isolated using precipitation in cold diethyl ether (3×) and was obtained as a white solid with a 93% isolated yield. Figure 2b illustrates the 500 MHz ^1^H NMR spectrum of alkyne-functionalized PEG-*b*-PCL (**2**) in CDCl_3_.

### 2.5. Synthesis of 6-Azidohexyl 4-Formylbenzoate (**3**)

The synthesis of 6-azidohexyl 4-formylbenzoate (**3**) was conducted following the two-step procedure reported by Yu et al. [32]. The structure of **3** was confirmed using 500 MHz ^1^H NMR analysis in CDCl_3_, as shown in Figure 2c.

### 2.6. Synthesis of PEG-b-PCL-g-ALD (**4**)

**Figure 3:**
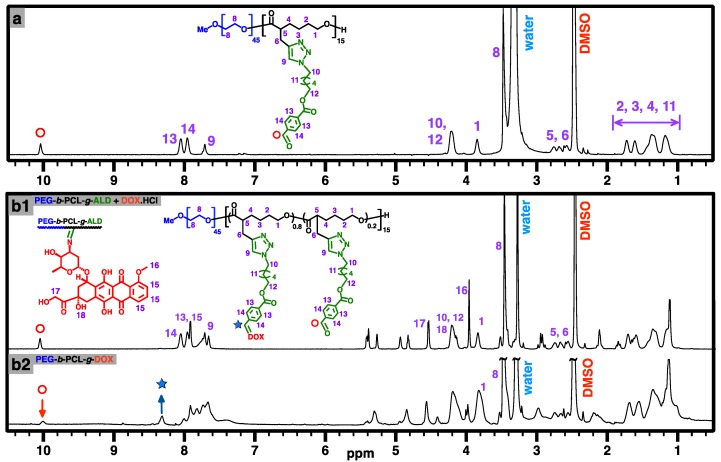
500 MHz ^1^H NMR spectra: (**a**) PEG-*b*-PCL-*g*-ALD (**4**), (**b1**) mixture of PEG-*b*-PCL-*g*-ALD and DOX.HCl, and (**b2**) PEG-*b*-PCL-*g*-DOX (**5**) in DMSO-D_6._

The synthesis of PEG-*b*-PCL-*g*-ALD (**4**) was conducted through CuAAC click chemistry. In a 10-mL Schlenk flask, CuSO_4_.5H_2_O (2.2 mg, 8.7 µmol), _L_-ascorbic acid sodium salt (NaAsc) (4.2 mg, 21 µmol), **3** (53 mg, 193 µmol, 1.1 equiv. per alkyne group), and **2** (50 mg, 11.6 µmol, with 175 µmol of alkyne group) were added under N_2_. Dried DMF (2 mL) was added via a nitrogen-purged syringe. The mixture was then stirred for 30 min to dissolve all the reagents, followed by five freeze-pump-thaw cycles. The reaction was allowed to continue for 2 days and then stopped by bubbling with air for 1 h. The copper content of the reaction mixture was removed by passing the crude through a small basic alumina column. Finally, the eluent was precipitated in cold diethyl ether (Et_2_O) to obtain **4** as the final product. The structure of **4** was confirmed using 500 MHz ^1^H NMR analysis, as shown in Figure 3a.

### 2.7. Synthesis of PEG-b-PCL-g-DOX (5)

The synthesis of PEG-*b*-PCL-*g*-DOX (**5**) was achieved through a Schiff base conjugation reaction between **4** and DOX. In a 10-mL Schlenk flask wrapped in aluminum foil, DOX.HCl (36 mg, 61.8 µmol) was dissolved in 1 mL of dried DMSO. TEA (12.5 mg, 123.6 µmol, 2 equiv. relative to DOX) was air-free added to the flask. The mixture was stirred at room temperature for 3 h, during which the HCl moiety of DOX.HCl was reacted and resulted in neutral DOX. The neutral DOX solution was then added dropwise to another 10-mL Schlenk flask containing a solution of **4** (35 mg, 4.12 µmol, with 61.8 µmol of the aldehyde group, 1 equiv. relative to DOX) in 1 mL of dried DMSO. The reaction was stirred at 30 °C for 3 days under N_2_, and aliquots were removed for ^1^H NMR analysis via a nitrogen-purged syringe. After the desired conversion was achieved, the reaction was stopped, and the reaction solution was dialyzed in THF for 3 days, using a dialysis membrane with MW cut-off (MWCO) of 3.5 kDa. The resulting solution was then evaporated to dryness in vacuo to give PDC **5** as a red solid. Figure 3b shows a 500 MHz ^1^H NMR spectrum of **5** in DMSO-D_6_.

### 2.8. DOX Release Study

PDC **5** were dissolved in 2.0 mL of PBS buffer (0.5 mg/mL) at pH 7.4 and pH 5.5, respectively. Each solution was then transferred to a dialysis bag (MWCO: 3.5 kDa), followed by incubation in 50 mL of the same buffer medium at 37 °C. To measure the amount of drug released, every time a 5 mL sample was withdrawn from the exterior buffer medium for UV-VIS analysis. The concentration of DOX in each sample was calculated based on a standard curve that was obtained from a series of DOX solutions with predetermined concentrations.

### 2.9. Cell Culture

MCF-7 human breast cancer cells were purchased from American Type Tissue Collection (ATTC, Manassas, VA, USA). The cells were cultured in Dulbecco’s modified Eagle’s medium (DMEM, Thermo Fisher Scientific, 11965-092) supplemented with 10% fetal bovine serum (FBS; Thermo Fisher Scientific, 26140079) and 1% penicillin-streptomycin (PS; Life Technologies, 15140-122). The cells were passaged every two or three days and maintained at 37 °C with 5% CO_2_.

### 2.10. In Vitro Cytotoxicity Assay

For the cytotoxicity assessment of free DOX, PEG-*b*-PCL-*g*-ALD (**4**), and PEG-*b*-PCL-*g*-DOX (**5**) in MCF-7 breast cancer cells, the cells were first seeded in 96-well plates (Greiner Bio-one; 655180) at the density of 1 × 10^4^ cells per well. After overnight incubation at 37 °C, the cells were treated with vehicle control, free DOX, PEG-*b*-PCL-*g*-ALD (**4**), and PEG-*b*-PCL-*g*-DOX (**5**) with different concentrations. At 24 h post-treatment, the cell viability was measured using alamarBlue assay following the manufacturer’s protocol. Briefly, one part of the alamarBlue reagent was mixed with ten parts of the DMEM medium. Then the alamarBlue/DMEM mixture was incubated with cells at 37 °C for 3 h, protected from light. The fluorescence intensity of each well was measured using a TECAN microplate reader (San Jose, CA, USA) with the excitation and emission wavelengths at 560 nm and 590 nm, respectively. The cell viability was normalized to the negative control, i.e., cells treated with vehicle control.

### 2.11. Cellular Uptake Assay and Confocal Imaging

For cellular uptake studies, MCF-7 cells were seeded in 6-well plates (Greiner Bio-one, 657160) at the seeding density of 2 × 10^5^ cells per well and allowed to grow overnight. The cells were treated with vehicle control, free DOX, and PEG-*b*-PCL-*g*-DOX (**5**) at a DOX concentration of 1 µg/mL. The cells were collected at 24 h post-treatment and fixed with 4% paraformaldehyde (Acros, 41678-5000). A BD Fortessa flow cytometer (BD bioscience, San Jose, CA, USA) was used to measure the fluorescence intensities in the fluorescein isothiocyanate (FITC) channel (λ_ex_ = 490 nm, λ_em_ = 525 nm). For each sample, 10000 events were collected. The fluorescence signal was normalized to vehicle control and reported as mean ± SD. For confocal microscopy imaging, cells were mounted on glass slides directly and observed under an LSM 710 confocal microscope (ZEISS, Dublin, CA, USA). The fluorescence signals from DOX were detected with the excitation and emission wavelengths at 458 nm and 584 nm, respectively.

## 3. Results and Discussion

### 3.1. Synthesis

**Scheme 2:**
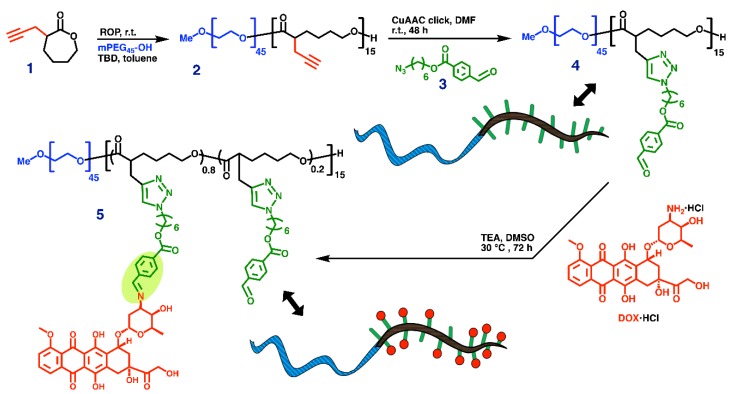
Synthesis of PEG-*b*-PCL-*g*-DOX (**5**).

**Figure 4:**
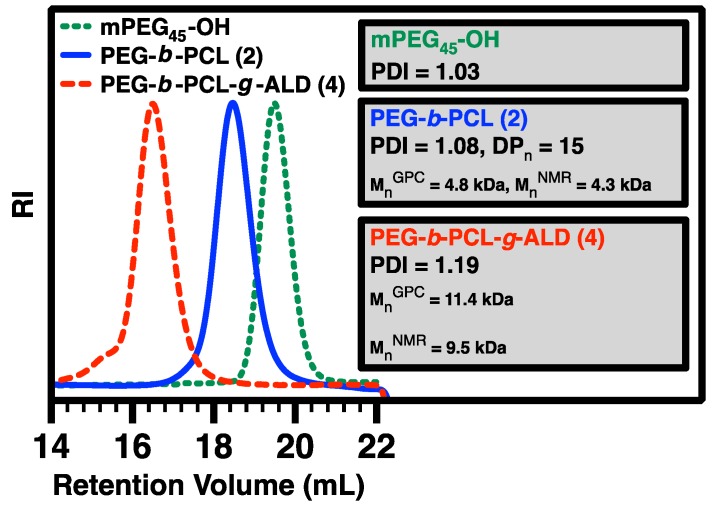
Gel permeation chromatography (GPC) characterization of mPEG_45_-OH as the ring-opening polymerization (ROP) macroinitiator, alkyne-functionalized PEG-*b*-PCL (**2**), and PEG-*b*-PCL-*g*-ALD (**4**) in DMF as eluent. The inserted panels show additional information about each polymer.

Alkyne-functionalized CL monomer **1** was synthesized in a 45% yield following a previously reported approach (Scheme 1) [16]. The structure of **1** was confirmed using 500 MHz ^1^H NMR analysis (Figure 2a). The multistep synthesis of the biodegradable PEG-*b*-PCL-*g*-DOX PDC (**5**) is illustrated in Scheme 2. Alkyne-functionalized PEG-*b*-PCL (**2**) was synthesized using ROP of **1** with mPEG_45_-OH (MW ~ 2 kDa) as the macroinitiator and TBD as the organocatalyst, following the method that has been reported previously [17,23,33]. The incorporation of PEG_45_ in the diblock copolymer is to create stealth shielding and promote the water solubility of the final micelle structure. The ROP process was conducted at room temperature ([mPEG-OH_45_]_0_:[**1**]_0_:[TBD]_0_ = 1:70:5) in dry toluene. The reaction stopped after the desired conversion was achieved as monitored using ^1^H NMR spectroscopy. The addition of a weak acid, i.e., acetic acid, in the reaction mixture resulted in deactivation of the catalyst and termination of the reaction. Prompt precipitation of the reaction solution in cold diethyl ether was very efficient, resulting in ~93% isolated yield of **2**. It should be noted that hexane can also be used for the precipitation purification process, leading to a high isolated yield. The structure of **2** was verified using 500 MHz ^1^H NMR analysis, as shown in Figure 2b. The resonance intensity at 2.0 ppm was assigned to the proton of alkyne functionality on the PCL-based block. The ^1^H NMR result confirmed ~22% of monomer conversion and a number-average degree of polymerization (DP_n_) of 15 based on the comparison of the resonance intensity of protons from the PEG block at 3.6 ppm with that of CH_2_O protons from the PCL block at 4.1 ppm. GPC analysis was used to characterize the polydispersity index (PDI) of the polymer formed using the ROP process (Figure 4). A low PDI of 1.08 was obtained for **2** by analyzing GPC data with a calibration using linear polystyrenes standards. It is important to maintain a low PDI of polymer for drug delivery applications because it can lead to polymer assemblies with narrow size distribution and consistent physicochemical properties.

CuAAC click reaction of alkyne-functionalized PEG-*b*-PCL (**2)** with azide-functionalized benzyl aldehyde (**3)** was performed to yield PEG-*b*-PCL-*g*-ALD (**4)**. Compound **3** was prepared following a previously reported method, and its structure was confirmed using ^1^H NMR analysis (Figure 2c) [31,32]. In the click reaction, molar ratios of [alkyne from **2**]_0_:[N_3_ from **3**]_0_:[CuSO_4_.5H_2_O]_0_:[NaAsc]_0_ = 1:1.1:0.05:0.125 were used with dried DMF as the solvent. After five freeze-pump-thaw cycles to remove oxygen, the reaction mixture was stirred for 48 h at room temperature. Then, the reaction was quenched by bubbling with air to promote the oxidization of Cu(I) to Cu(II). Purified **4** was obtained by passing the reaction mixture through a short basic alumina column to remove copper salts, followed by precipitation in cold diethyl ether to remove other impurities. The 500 MHz ^1^H NMR spectrum of **4** is shown in Figure 3a. At the same resonance position as the aldehyde proton from **3** at ~10 ppm (Figure 2c), a broader single resonance peak of aldehyde protons of **4** was observed (Figure 3a), indicating that the post-polymerization functionalization reaction was successful. Moreover, the presence of the resonance peak of triazole protons at ~7.7 ppm in Figure 3a further confirmed the occurrence of CuACC click reaction.

It should be noted that there are two possible approaches to synthesize the final PDC: (1) conjugating the spacer to the polymer and then reacting the resulting polymer with DOX, (2) conjugating the polymer to the presynthesized prodrug which is obtained from Schiff base reaction of **3** and DOX. Here, the first approach is preferred because it needs only one purification step of the DOX-containing molecule, and the prodrug purification step required for the other approach can be challenging. To prepare PDC from DOX and **4**, DOX.HCl needs to be neutralized at first by reacting with an excess amount of TEA base. This reaction was conducted in dried DMSO and at room temperature for 3 h ([DOX.HCl]_0_:[TEA]_0_ = 1:2), and the product of the reaction (i.e., free neutral DOX) was used directly to react with the aldehyde group of **4** ([aldehyde group from **4**]_0_:[DOX]_0_ = 1:1). Figure 3b1 illustrates the 500 MHz ^1^H NMR spectrum of the mixture of **4** and DOX. The resonance intensity at around 10.0 ppm corresponds to protons of aldehyde groups of **4** as labeled with a red circle. The reaction mixture was stirred in the dark at 30 °C for 3 days, with the reaction conversion monitored using ^1^H NMR analysis. Then the solution of the crude product was dialyzed (MWCO: 3.5 kDa) against THF for 3 days to remove unreacted DOX, TEA, and TEA·HCl salt gradually. The finally obtained product, i.e., PDC **5**, was characterized using 500 MHz ^1^H NMR analysis (Figure 3b2). The emergence of a resonance peak for benzyl imine protons (labeled with a blue star) at ~8.3 ppm, together with the reduction in resonance intensity of aldehyde protons at ~10.0 ppm, denotes the formation of PDC. As a diblock PDC, **5** possesses a hydrophilic block of mPEG_45_ with a molecular weight of ~2 kDa and a DOX-containing PCL-based block with a molecular weight of ~13.5 kDa. Due to the amphiphilic nature of **5**, micellar nanostructures were formed by dissolving the PDC in water without using an additional solvent.

### 3.2. Characterization

PEG-*b*-PCL-*g*-DOX PDC (**5**) was characterized systematically to reveal its structural features. According to the ^1^H NMR result (Figure 3b), ~80% of the aldehyde moieties on **4** was reacted to form PDC. This quantification was based on the comparison of resonance intensities of protons of newly formed benzyl imine linkages at ~8.3 ppm and the protons of mostly consumed aldehyde groups at ~10 ppm. Our previous study demonstrated that conjugation efficiency in a Schiff base reaction depends on reaction conditions of a specific reaction system, and conversions of 55% and 100% relative to DOX amount were reached by reactions of DOX with aldehyde-functionalized PLA for 24 h at room temperature using one and two equiv. of aldehyde relative to DOX, respectively [31,32]. In the current study of the Schiff base reaction of DOX with PEG-*b*-PCL-*g*-ALD (**4**), the conversion of 80% was achieved with one equiv. of the aldehyde of **4** relative to DOX, presumably because the reaction kinetics was promoted by using longer reaction time (3 days) and higher reaction temperature (30 °C). Because polymer micelle formation requires balanced hydrodynamic sizes of hydrophilic and hydrophobic blocks and the hydrophilic PEG_45_ block (MW ~ 2 kDa) of the diblock PDC is relatively short, higher DOX conjugation that can lead to an even larger hydrodynamic size of DOX-conjugated hydrophobic PCL-based block was not pursued in this work.

PDI values of polymers involved in the multi-step synthesis were determined using GPC (Figure 4). The macroinitiator mPEG_45_-OH showed a very low PDI of 1.03. Prepared using the ROP process, alkyne-functionalized PEG-*b*-PCL (**2**) exhibited a GPC curve, maintaining a low PDI of 1.08, but shifting to the high MW side as compared to mPEG_45_-OH. Such a low PDI of **2** supports the living characteristic of the ROP process catalyzed using TBD. The number-average molecular weight obtained from GPC (M_n_^GPC^) of **2** relative to linear polystyrene standards was 4.8 kDa, while the M_n_ value of **2**, according to NMR analysis (M_n_^NMR^) was 4.3 kDa. M_n_^NMR^ is considered more accurate, while M_n_^GPC^ may be overestimated because **2** is different from the linear polystyrene standards used in GPC calibration regarding the relationship between MW and hydrodynamic volume.

**Figure 5:**
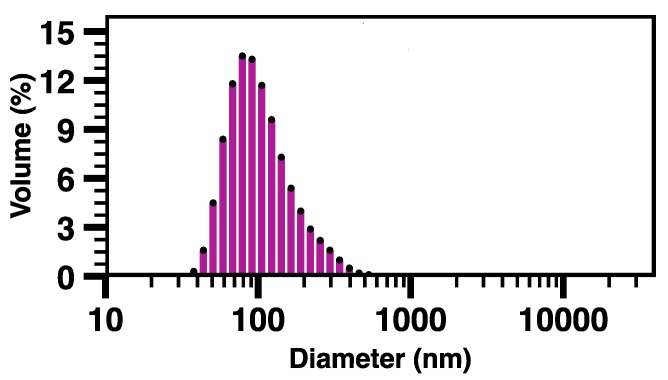
Volume-average size distribution for the PDC (**5**) micelles measured using dynamic light scattering (DLS).

**Figure 6:**
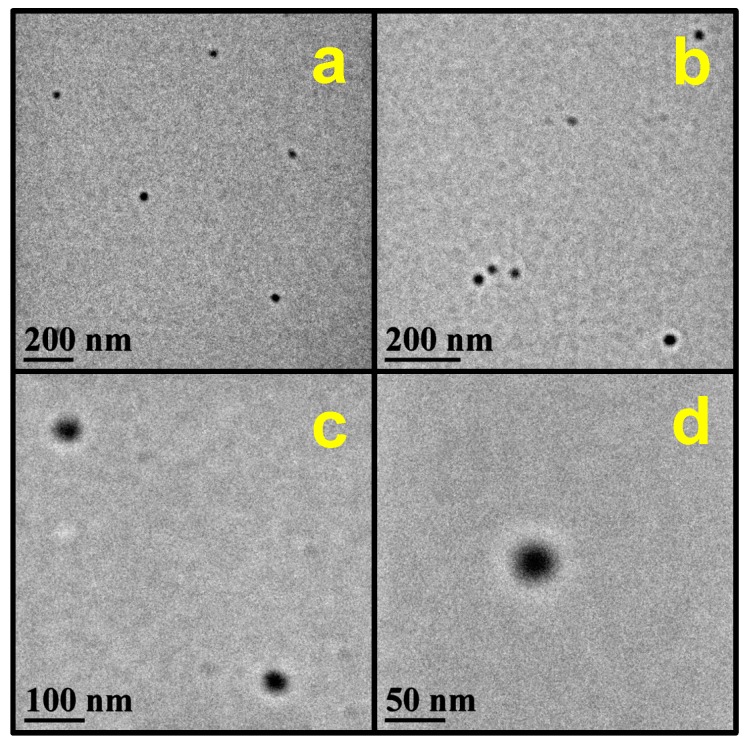
(**a**–**d**) TEM images of the PDC (**5**) micelles from aqueous solutions. TEM samples were prepared without staining.

The dimension of the PDC micelles formed from the self-assembly of **5** in aqueous solution was characterized using DLS. Figure 5 illustrates the volume-average size distribution of the micelles, indicating a volume-average hydrodynamic diameter (D_h,v_) of 128 ± 2 nm and a polydispersity of 0.23 for the PDC micelles. The nanostructure of the PDC micelles was characterized using transmission electron microscopy (TEM) according to our previously published sample preparation method [34]. The micelles showed high image contrast without applying any staining agent during TEM sample preparation (Figure 6). According to TEM images, the micelles exhibited sizes of ~60 nm at the dried state. The PEG shells of micelles appeared to have a much lighter grayscale color than the core domains consisting of the DOX-containing PCL-based block in the TEM images. The TEM size of the micelles is smaller than the DLS size because the diameter of micelles would shrink from a hydrated state to a dried state.

### 3.3. Drug Release Study

**Figure 7:**
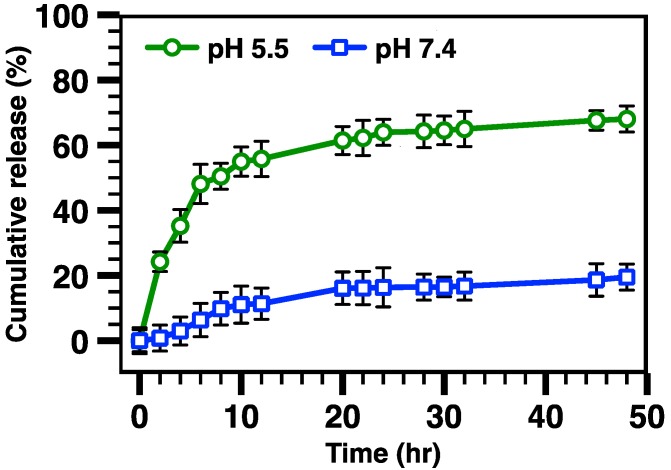
DOX release profiles from the PDC (5) micelles at pH 5.5 and 7.4 at 37 °C. Each data points with error bars represent the mean value ± the standard deviation obtained from three independent measurements.

Drug release behavior is one of the essential characteristics of drug delivery systems. Accordingly, an in vitro DOX release study from PEG-*b*-PCL-*g*-DOX PDC **5** was conducted using a dialysis approach. Two parallel experiments in PBS buffer were conducted at 37 °C, one at physiological pH (7.4) and the other at acidic pH (5.5). The pH 5.5 was selected in order to demonstrate pH-responsive DOX release from **5**, because tumor tissue and intracellular endosome and lysosome of cancer cells possess an acidic environment [32]. According to Figure 7, the DOX release was considerably faster at pH 5.5 than pH 7.4. While only around 20% of DOX was released in pH 7.4 over the period of 48 h, 50% of DOX release was observed at pH 5.5 just after 6 h. This faster DOX release at pH 5.5 can be attributed to the acid-labile benzyl imine Schiff base linkage, which is highlighted with light green in the structure of **5** in Scheme 2.

### 3.4. Cytotoxicity and Cell Internalization Studies

**Figure 8:**
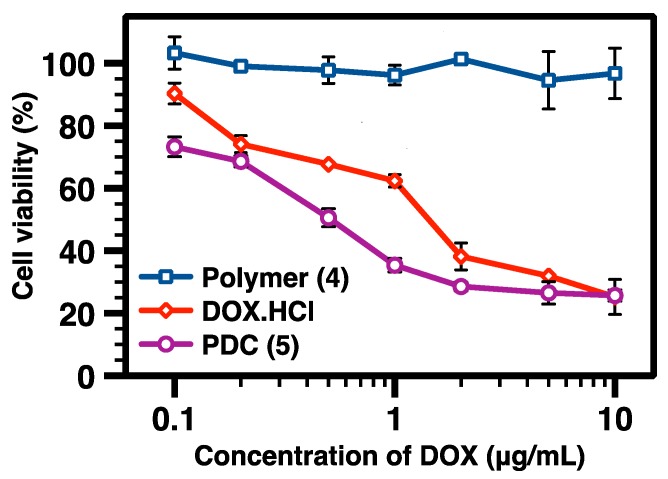
Cytotoxicity of PEG-*b*-PCL-*g*-ALD polymer (**4**), DOX·HCl, and PEG-*b*-PCL-*g*-DOX PDC (**5**) against MCF-7 cells after 24 h incubation. Each data point with error bars represents the mean value ± the standard deviation obtained from three independent measurements. Polymer (**4**) was used as the same molar concentration of PDC (**5**).

Cytotoxicity of PEG-*b*-PCL-*g*-DOX **5** relative to free DOX and PEG-*b*-PCL-*g*-ALD **4** was evaluated using alamarBlue cell viability assay against MCF-7 breast cancer cells after 24 h of incubation (Figure 8). The precursor polymer **4** demonstrated no considerable cytotoxicity over a broad range of concentrations. On the other hand, both DOX and **5** exhibited significant cytotoxicity, which increased with the increase of DOX concentration. Specifically, the IC_50_ (the concentration of DOX required to kill 50% of cells) of **5** (0.50 μg/mL) was found to be only 1/3 of the IC_50_ of free DOX (1.5 μg/mL). Overall, **5** resulted in higher anticancer therapeutic efficacy than free DOX as the curve of **5** was always below that of free DOX.

**Figure 9:**
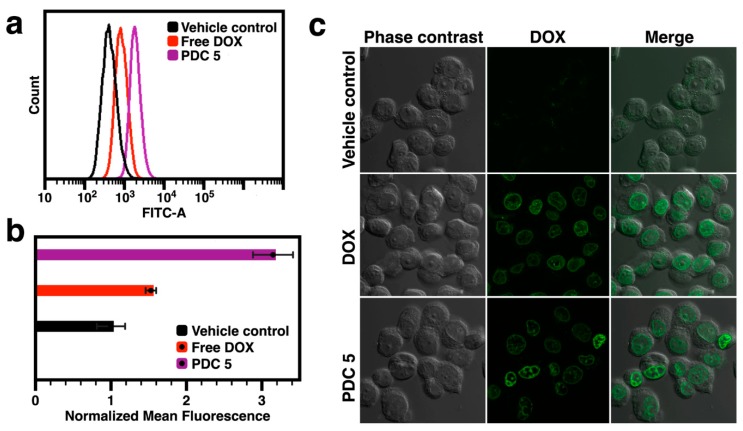
(**a**) A typical flow cytometry data set showing cellular uptake of free DOX and PDC **5** by MCF-7 cells after 24 h incubation; (**b**) mean fluorescence intensity of DOX obtained from flow cytometry. For each data point, the error bar represents standard deviation resulting from three independent experiments; (**c**) confocal microscopy images of MCF-7 cells after 24 h incubation with DOX and PDC **5**.

Cellular uptake of **5** was studied using both flow cytometry and confocal microscopy, with MCF-7 cells as the model cancer cells. As shown in Figure 9a, the fluorescence signal from the cells treated with PDC **5** was much stronger than the cells treated with free DOX. Figure 9b revealed that the mean fluorescence intensity of the cells treated with PDC **5** were ~2 folds of free DOX-treated cells and ~3 folds of cells treated with vehicle control, respectively. As illustrated in Figure 9c, confocal microscopy imaging also confirmed that DOX-containing PDC **5** was taken up effectively by the cells and mainly accumulated in the nucleus. The facilitated DOX internalization via PDC **5** may be the key factor for the higher cytotoxicity of **5** relative to free DOX, as observed in the cytotoxicity study.

## 4. Conclusions

In summary, well-defined PEG-*b*-PCL-*g*-DOX PDC (**5)** was successfully synthesized and subsequently tested for anticancer efficacy against MCF-7 breast cancer cells. Alkyne-functionalized CL (**1)** as the monomer of the PCL-based block was prepared and then polymerized through ROP to form alkyne-functionalized PEG-*b*-PCL (**2)**. mPEG_45_-OH was used as the macroinitiator, to promote water-solubility and to provide stealth properties of the final PDC. Because aldehyde functionalities were needed for the formation of pH-responsive PDC with benzyl imine Schiff base linkage, CuAAC click chemistry was used for the reaction between alkyne-functionalized **2** and azide/aldehyde-functionalized **3** to form PEG-*b*-PCL-*g*-ALD (**4**). Finally, Schiff base reaction of **4** with DOX yielded the amphiphilic diblock PDC. The PDC and PDC micelles were comprehensively characterized. Acid-sensitive DOX release from the PDC micelles was confirmed. Cytotoxicity and cellular uptake studies showed that the PDC micelles could be readily taken up by MCF-7 cells to result in enhanced therapeutic effects as compared to free DOX. Overall, the results of this work indicate both preparation feasibility and promising application potentials of PDC micelles as novel drug delivery systems.
